# Physiological reactivity to acute mental stress in essential hypertension—a systematic review

**DOI:** 10.3389/fcvm.2023.1215710

**Published:** 2023-08-11

**Authors:** Lisa-Marie Walther, Petra H. Wirtz

**Affiliations:** ^1^ Biological Work and Health Psychology, University of Konstanz, Konstanz, Germany; ^2^ Centre for the Advanced Study of Collective Behaviour, University of Konstanz, Konstanz, Germany

**Keywords:** essential hypertension, physiological stress reactivity, acute mental stress, systematic review, stress

## Abstract

**Objective:**

Exaggerated physiological reactions to acute mental stress (AMS) are associated with hypertension (development) and have been proposed to play an important role in mediating the cardiovascular disease risk with hypertension. A variety of studies compared physiological reactivity to AMS between essential hypertensive (HT) and normotensive (NT) individuals. However, a systematic review of studies across stress-reactive physiological systems including intermediate biological risk factors for cardiovascular diseases is lacking.

**Methods:**

We conducted a systematic literature search (PubMed) for original articles and short reports, published in English language in peer-reviewed journals in November and December 2022. We targeted studies comparing the reactivity between essential HT and NT to AMS in terms of cognitive tasks, public speaking tasks, or the combination of both, in at least one of the predefined stress-reactive physiological systems.

**Results:**

We included a total of 58 publications. The majority of studies investigated physiological reactivity to mental stressors of mild or moderate intensity. Whereas HT seem to exhibit increased reactivity in response to mild or moderate AMS only under certain conditions (i.e., in response to mild mental stressors with specific characteristics, in an early hyperkinetic stage of HT, or with respect to certain stress systems), increased physiological reactivity in HT as compared to NT to AMS of strong intensity was observed across all investigated stress-reactive physiological systems.

**Conclusion:**

Overall, this systematic review supports the proposed and expected generalized physiological hyperreactivity to AMS with essential hypertension, in particular to strong mental stress. Moreover, we discuss potential underlying mechanisms and highlight open questions for future research of importance for the comprehensive understanding of the observed hyperreactivity to AMS in essential hypertension.

## 1. Introduction

Essential hypertension (EHT), the chronic elevation of blood pressure (BP) without secondary causes, is one of the most prevalent risk factors for cardiovascular disease (CVD) (1, 2). Exaggerated physiological reactions to acute stress (AMS), or more precisely acute mental stress, have been proposed to be involved in mediating the CVD risk with hypertension (3, 4). Specifically, several lines of evidence suggest that exaggerated mental stress-induced cardiovascular and neuroendocrine reactions predict not only premature development of hypertension and other precursors of coronary heart disease, but also an accelerated progression of atherosclerosis and the likelihood of having a future acute coronary syndrome such as myocardial infarction (3–6). In addition to cardiovascular and neuroendocrine hyperreactivity, heightened stress reactivity of intermediate biological CVD risk factors (e.g., blood lipids and lipoproteins, hemostatic factors, inflammatory activity) may play an important role in hypertension and hypertension development (7–9) as well as in the interface between stress and heightened risk for CVD morbidity and mortality (10–13). Since the 1970s, a variety of studies have compared the physiological reactivity to AMS in essential hypertensive individuals (HT) and normotensive individuals (NT) applying mental stressors of different severity and considering different stress-reactive physiological systems. However, a systematic summary of the literature is lacking so far. Here, we therefore summarize for the first time the hitherto published evidence on physiological reactivity to AMS in HT as compared to NT regarding the primary endocrine stress-axes, i.e., the sympathetic-adrenal-medullary (SAM) axis, the hypothalamus-pituitary-adrenal (HPA) axis, and the renin-angiotensin-aldosterone system (RAAS), in addition to major stress-reactive intermediate biological risk factors for CVD (coagulation, blood lipids and lipoproteins, and inflammatory measures). Based the hitherto reported cardiovascular hyperreactivity in EHT to different psychological and physiological stressors including AMS [for review see: (14, 15)] on the one hand, and the interrelation of stress-reactive physiological systems (16–20) on the other hand, we hypothesized a generalized physiological hyperreactivity to AMS in EHT across different stress-reactive physiological systems. Moreover, we discuss potential mechanisms underlying the obtained summarized stress reactivity findings in EHT.

### 1.1. Essential hypertension as a major CVD risk factor

Arterial hypertension is a highly prevalent medical condition defined as a chronic elevation of systolic BP (SBP) ≥140 mmHg, a chronic elevation of diastolic BP (DBP) ≥90 mmHg, or both (21). The worldwide prevalence of hypertension among adults is about 30% with noticeable but stable differences across countries and with a steep increase with ageing (21, 22). Hypertension presents a major if not the greatest risk factor for CVD (23). With BP increase from normal to severe levels, the risk for accelerated atherosclerosis, coronary heart disease, left-ventricular hypertrophy, and stroke increases markedly (24, 25). Moreover, high BP was identified to be the leading single risk factor globally in 2015 accounting for 10.7 million deaths (26). Intriguingly, most individuals diagnosed with arterial hypertension suffer from EHT, also referred to as primary, idiopathic, or systemic hypertension, meaning that there is no medical cause for their condition (27).

### 1.2. Acute mental stress

According to Lazarus & Folkman (28), stress is the result of cognitive appraisal processes concerning the interaction between the individual and the environment. More precisely, stress occurs when an individual experiences demands or threats, i.e., encounters a so-called *stressor*, without sufficient resources to meet these demands or mitigate the threats. This perceived imbalance between situational demands and personal resources triggers *stress responses* comprising a wide variety of psychophysiological reactions in order to counteract the stressful circumstances (29). Stress can occur acutely, i.e., lasting for minutes to hours (i.e., acute stress) or chronically, i.e., persisting for days to months (i.e., chronic stress) (30). In human acute stress research, most studies focus on AMS. Mental stressors commonly used comprise cognitive tasks, public speaking tasks (PST), and the combinations of cognitive and PST (31, 32). Typical *cognitive tasks* are mental arithmetic tasks (MA), Raven's progressive matrices (33), general knowledge quizzes, visual puzzles, and interference tasks such as the mirror tracing task or the Stroop color word interference task (SCWT) (34). In *PST*, participants are instructed to prepare and deliver a short speech in front of an audience on an assigned, often controversial topic. Notably, cognitive tasks have been shown to induce physiological stress reactions of minor intensity as compared to PST (35). Strongest physiological stress reactions have been observed in reaction to the combination of cognitive tasks with PST (31). Central for induction of strong stress reactions is the combination of social-evaluative threat (i.e., potential negative evaluation of the self, e.g., by an evaluative audience) and uncontrollability (i.e., perception that avoidance of negative consequences, termination of aversive experiences or succession is not or will not be possible, e.g., false feedback or time-pressure) (31). A frequently used psychosocial stressor that combines PST and MA is the Trier Social Stress Test [TSST (36)]. The TSST comprises a short introduction phase followed by a 3-min preparation phase, a 5-min mock job interview, and a 5-min MA in front of an audience with video and audio recording. Also, the Montreal Imaging Stress Task [MIST (37)] that comprises a series of computerized mental arithmetic challenges, was initially developed for inducing AMS in a functional imaging setting and includes social evaluation and uncontrollability (31).

Given these differences in stressor potency to elicit physiological stress reactions, we discriminate between reactivity to mild, moderate, and strong mental stressors. We refer to cognitive tasks as mild, to PST as moderate, and to the TSST and the MIST (that both combine PST with MA) as strong mental stressors.

### 1.3. Major stress-reactive physiological systems

Following stress encounter, multiple interrelated physiological systems are activated, comprising neuroendocrine stress axes, i.e., the SAM axis, the HPA axis, and the RAAS, as well as stress-reactive intermediate biological risk factors for CVD.

The first activated stress-reactive system is the SAM axis (38). Here, stress perception activates preganglionic sympathetic neurons in the intermediolateral cell column of the spinal cord. This subsequently leads on the one hand the activation of end organs via direct sympathetic signaling pathways where norepinephrine (NEP) acts as postganglionic transmitter and on the other hand to stimulation of the secretion of the catecholamines NEP and epinephrine (EP) from chromaffin cells of the adrenal medulla into the circulation (39). Both, the NEP released directly at sympathetic synapses and the circulating catecholamines released from the adrenal medulla, induce rapid physiological adaptions in order to allow to “fight-or-flight”, e.g., increases of BP, heart rate (HR), or vasodilation of central blood vessels (e.g., in skeletal muscles, heart, and brain) (38). Measures of acute *SAM axis* reactivity comprise on the endocrine level the plasma catecholamines NEP and EP, on the cardiovascular level BP, HR, cardiac output, stroke volume, or total peripheral resistance. Also, the enzyme salivary alpha amylase (sAA), secreted from the salivary glands under sympathetic stimulation, is considered a surrogate marker of catecholamine release, in particular of NE (40). Notably, all these parameters reach their maximum stress reactivity during or immediately after stress. Recovery to baseline levels can be observed within 5–20 min after stress cessation for most parameters except for BP where delayed recovery periods have been reported (41–44).

Simultaneously to the sympathetic activation, the activity of the *parasympathetic nervous system (PNS)* as counterpart of the SNS, predominant under quiet, resting conditions, decreases. Parasympathetic (re)activity can be assessed via heart rate variability (HRV) reflecting beat-to-beat changes in RR intervals (45, 46). HRV parameters reflecting predominantly PNS (re)activity comprise the square root of the mean of the sum of the squares of differences between adjacent normal-to-normal interval’ (RMSSD), the percentage difference between adjacent normal-to-normal intervals that are greater than 50 ms (PNN50), and the high frequency (HF) band (0.15–0.40 Hz) (45, 46). Notably, given that HRV depends on resting heart rate as well as the frequency and depth of respiration, reliable HRV assessment requires adjustment for these potentially influencing factors (47). In reaction to AMS, immediate significant decreases in RMSSD, pNN50, and HF have been observed in healthy individuals (48–50).

In addition the activation of the SAM axis, perception of stress activates hypophysiotropic neurons in the paraventricular nucleus of the hypothalamus that lead to the release of the hypothalamic hormones, corticotropin-releasing hormone (CRH) and arginine vasopressin (AVP), which are the principal regulators of the *HPA axis* (39). CRH and AVP stimulate the secretion of adrenocorticotropin hormone (ACTH) from the anterior pituitary (51). ACTH primarily acts on the zona fasciculata of the adrenal cortex, where it initiates the synthesis and secretion of cortisol (52). Cortisol acts on intracellular cytosolic glucocorticoid and mineralocorticoid receptors throughout the entire body, in order to prepare the body for exposure to stress (53). In human stress research, assessment of acute HPA axis (re)activity usually comprises peripheral ACTH and/or cortisol measurements (54, 55). While ACTH is usually measured from blood plasma, cortisol can reliably be measured from both, blood plasma and serum (bound and free fractions of cortisol), as well as from saliva (biologically active free fraction of cortisol) (56, 57). With respect to stress reactivity, stress exposure by TSST induces fast increases in ACTH that peak immediately after TSST cessation and return to baseline levels about 40 min after stress cessation (36, 58). Salivary cortisol shows a comparably delayed stress reactivity with highest levels peaking about 30 min after TSST beginning, or about 10 min after its cessation, respectively, that last for another 10–20 min and then slowly return to baseline levels about 60 min after stress cessation (36, 59). Reactivity of plasma and serum cortisol starts a bit earlier but the subsequent reactivity pattern is comparable (36, 59).

Both, the SAM axis and the HPA axis, can interact with the *RAAS*. With respect to the SAM axis, catecholamines released from the adrenal medulla induce the synthesis and secretion of renin via β-adrenergic receptors on juxtaglomerular cells in the kidney. Subsequently, renin catalyzes the hydrolysis of angiotensinogen produced in the liver and fat tissues to angiotensin-I (ANG-I) (60). ANG-I is transported to lung capillaries, endothelial cells, and kidney endothelial cells via the blood circulation where it is converted to angiotensin-II (ANG-II) by angiotensin-converting enzyme (ACE) (61). ANG-II then stimulates the production of aldosterone in the zona glomerulosa of adrenal cortex (62). With respect to the HPA axis, the stress-induced release of ACTH stimulates in addition to the production of cortisol in the zona fasciculata also the production of aldosterone in the zona glomerulosa of the adrenal cortex (60). Parameters indicative of RAAS activation comprise plasma renin and plasma renin activity (PRA), ANG-II, and aldosterone that can be measured in blood plasma. Notably, we recently showed that aldosterone can also be validly assessed from saliva (18). While plasma renin measures and ANG-II increased immediately after stress with quick return to baseline levels, highest levels of both, plasma and salivary aldosterone, were observed during the 20 min after TSST cessation (18, 63). Stress-induced aldosterone level increases returned to baseline within about 90–120 min after stress with respect to salivary aldosterone and within 180 min after stress for plasma aldosterone (18).

In addition to the activation of endocrine stress axes, AMS elicits increases in intermediate biological risk factors for CVD. Acute stress induces activation of *coagulation* molecules, platelets, and fibrinolysis resulting in net hypercoagulability (11, 64) that presumably aims at protecting the organism from lethal hemorrhage in case of a potentially resulting injury in fight-or-flight situations (65). Major clotting-related targets of stress-induced catecholamine-release include platelet activation, the hepatic release of clotting factors VII (FVII:C) and VIII (FVIII:C), as well as excretion of hemostatically active von Willebrand factor antigen (VWF) and the pro-fibrinolytic tissue-type plasminogen activator (t-PA) from endothelial storage pools into the circulation (66, 67). Notably, further stress-induced t-PA increases in the circulation origin from sympathetic innervated artery walls (68). Subsequently, various resulting processes involving the clotting factors IX (FIX:C), X (FX:C), XI (FXI:C), and XII (FXII:C), lead to further platelet activation and the formation of a critical amount of thrombin, initiating the conversion of fibrinogen to fibrin and clot formation (69). Termination of clot formation involves several anticoagulant mechanisms leading to inactivation of thrombin by formation of thrombin-antithrombin III (TAT) complexes (70). Laboratory studies in healthy humans show increased stress-induced activation of the clotting parameters FVII:C, FVIII:C, platelets, t-PA, TAT complexes, and fibrinogen with highest levels immediately after stress and recovery back to prestress levels about 20–45 min after stress (12, 66, 71, 72). Notably, stress reactivity kinetics of the fibrin cleavage product and hypercoagulability parameter D-Dimer are not fully understood yet with some studies reporting highest D-dimer levels immediately after stress and other study observing delayed increases (71, 73–75).

*Blood lipids and lipoproteins* represent further stress-reactive intermediate biological risk factors. Stress-induced changes in the prothrombotic lipid parameters total cholesterol (TC), triglycerides (TG), and low density lipoprotein-cholesterol (LDL-C) as well as the antithrombotic high density lipoprotein-cholesterol (HDL-C) presumably aim at providing energy for muscles activity in fight-or-flight responses (76). Studies investigating blood lipid and lipoprotein reactivity to AMS report stress-induced increases in prothrombotic and antithrombotic blood lipid parameters (13, 77–82). However, not all these studies controlled for stress-induced hemoconcentration as an important confounding factor (79). Notably, stress can induce transient acute loss of plasma volume into the extravascular space, which results in the concentration and passive increase of larger (>69 kDa) and thus non-diffusible blood constituents such as lipids (83, 84). After control for stress hemoconcentration effects on blood lipid reactivity (85), stress-induced increases in prothrombotic blood lipids and lipoproteins have been observed in reaction to mental stressors of moderate or strong intensity (13, 78, 79, 81) but not in reaction to mild mental stressors (79). With respect to HDL-C, results of studies controlling for stress hemoconcentration remain inconclusive (13, 78, 79). Stress hemoconcentration-independent changes in blood lipid parameters in reaction to AMS have been proposed to relate to stress-induced SNS activation (86, 87). With respect to the kinetics of lipid stress reactivity to moderate and strong mental stress, most studies point to highest levels of prothrombotic blood lipids and lipoproteins at or shortly after stress cessation (13, 78, 79, 81).

Finally, acute stress is capable of inducing *immune activation*. A recent systematic review and meta-analysis summarized the increasing number of studies investigating reactivity of inflammatory markers in reaction to AMS in healthy individuals (88). Robust stress-induced increases of moderate to large effect size were found for the cytokines interleukin (IL)-6 and IL-1β (88, 89), in addition to small to moderate stress-induced increases in tumor necrosis factor (TNF)-α (88). Stress-induced C-reactive protein (CRP) increases however could not be confirmed (88). It is assumed that the stress-induced proinflammatory response has evolved in order to protect an individual from potential immediate injury and infection due to stressors such as e.g., a predator attack (30, 90). Stress reactivity kinetics differ between cytokines [likely due to differences in half-lives or clearance mechanisms (91)] and are not fully understood yet given the investigation period of only up to 2 h in existing studies. Notably, inflammatory markers were found to increase delayed, i.e., not before 10 min after stress cessation. Peak levels of circulating IL-1β and TNF-α were mostly observed 40–50 min after stress, while peak levels of IL-6 occurred later, at 90–120 min after stress (88). The exact mechanisms underlying the stress-induced increases in inflammatory markers are beginning to be understood. It has been speculated that cytokine increases in reaction to acute stress are mediated via the SNS and intracellular proinflammatory activity (88, 89, 92). With respect to down-regulation or delay of the inflammatory response to acute stress, cortisol, and thus HPA axis reactivity, has been proposed to play an important role (88, 93–95).

## 2. Systematic review: physiological reactivity to AMS in essential hypertension

Exaggerated stress-induced physiological reactions have not only been associated with premature development of hypertension (6, 96) but also been proposed to play an important role in mediating the CVD risk with hypertension (3). In the following, we summarize the hitherto existing literature on the physiological reactivity to AMS with respect to the SAM and HPA axes, the RAAS as well as intermediate biological risk factors in HT compared to NT. In line with existing meta-analytic evidence for a cardiovascular hyperreactivity with EHT to different stressors including AMS (14) and the interrelation of stress-reactive physiological systems (16–20), we expect our systematic review to support a generalized physiological hyperreactivity to AMS with EHT across different stress-reactive physiological systems.

### 2.1. Methods

We performed a systematic review according to the “preferred reporting items for systematic reviews and meta-analysis guidelines” (PRISMA) (97). Our literature search was conducted in November and December 2022.

#### 2.1.1. Search strategy

In our main literature search on studies investigating physiological reactivity to AMS in manifest EHT, we conducted a systematic search on PubMed using the combinations of search terms “hypertension” AND (“mental stress” OR “psychological stress” OR “psychosocial stress” OR “acute stress”) together with the keywords of each of the considered stress-reactive physiological systems: the SNS (sympathetic reactivity, sympathetic nervous system, catecholamines, norepinephrine, epinephrine, cardiovascular reactivity, blood pressure, heart rate, salivary alpha-amylase), the PNS (parasympathetic reactivity, parasympathetic nervous system, heart rate variability, RMSSD, HF, pNN50), the HPA axis (HPA axis, CRH, ACTH, cortisol), the RAAS (renin-angiotensin-aldosterone-system, renin, angiotensin, aldosterone), the coagulation system (coagulation, procoagulant, d-dimer, fibrinogen, FVII, FVIII), blood lipids and lipoproteins (blood lipids, LDL, HDL, triglycerides, cholesterol), the immune system (inflammation, inflammatory marker, interleukin, t-PA, TNF-alpha, NF-kappaB, CRP). All identified publications were screened to exclude duplicates. Subsequently, both, abstracts and full texts of remaining publications were reviewed manually for formal and content-related inclusion criteria. We moreover manually searched the reference lists of included publications for further relevant publications not identified by the initial search.

**Figure 1:**
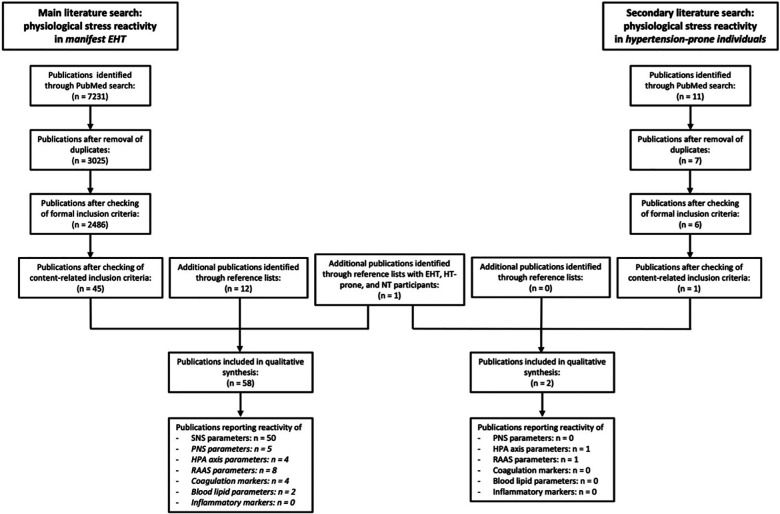
Flow diagram of the process of study selection. A secondary literature search was conducted for endocrine stress systems and biological risk factors with <10 publications (in italics) included in qualitative synthesis of main literature search. EHT, essential hypertension; SNS, sympathetic nervous system; PNS, parasympathetic nervous system; HPA axis, hypothalamus-pituitary-adrenal axis; RAAS, renin-angiotensin-aldosterone-system; *n* = number of publications.

Moreover, we *a-priori* defined that in case of less than 10 identified eligible publications for a stress-reactive physiological systems, our main literature search for this system will be extended to a secondary literature search on hypertension-prone individuals. Study participants were considered as hypertension-prone if at least one of their parents had EHT (parental hypertension) and/or if they were designated as hypertension-prone by the authors of the respective publication. Based on this definition, we repeated the previous systematic literature for all stress-reactive systems except the SNS (see Figure 1) by using the search terms “hypertension-prone” OR “parental hypertension” instead of “hypertension”.

#### 2.1.2. Eligibility and inclusion criteria

With respect to formal inclusion criteria, we considered original articles and short reports published in English language that were published in peer-reviewed journals. Meta-analyses, reviews, case reports, editorials, and conference abstracts were not considered. With respect to content-related inclusion criteria, we included studies reporting experimental data on human physiological reactivity in reaction to AMS assessed in essential hypertensive (main literature search) or hypertension-prone (secondary literature search) individuals of at least 18 years as compared to a normotensive control group. Acute mental stressors considered in this review comprise cognitive tasks (MA, Raven's progressive matrices, general knowledge quizzes, visual puzzles, mirror tracing tasks, SCWT) (mild mental stressors), PST (moderate mental stressors), and the combinations of cognitive and PST (strong mental stressors). Notably, publications that did not differentiate between reactivity to mental stressors and other stressor types were not further considered. Regarding the physiological reactivity assessment, publications had to capture at least one of the previously defined physiological parameters of interest (plasma EP, plasma NEP, BP, HR, sAA, RMSSDN, PNN50, HF, ACTH, plasma or salivary cortisol, plasma renin or plasma renin activity, ANG-II, aldosterone, any coagulation or inflammatory marker, or blood lipid parameter). Notably, some studies have resulted in multiple publications, addressing different physiological systems and parameters with respect to AMS reactivity in EHT. To prevent a resulting bias, we included the first published data of the respective physiological systems and parameters.

#### 2.1.3. Data collection and synthesis

We retrieved and tabulated the following data from publications with respect to each defined physiological parameter: participant characteristics, stressor characteristics, and findings with respect to physiological stress reactivity.

### 2.2. Results

With respect to the main literature search, our search yielded 2,486 potentially relevant publications after removal of duplicates and checking of formal inclusion criteria (see Figure 1). Ultimately, 45 publications met content-related inclusion criteria and 13 additional eligible publications were identified from reference lists, rendering a total of 58 publications included in the qualitative synthesis of literature on physiological reactivity to AMS in EHT. 50 publications report reactivity of SNS parameters, 5 of PNS parameters, 4 of HPA axis parameters, 8 of RAAS parameters, 4 of coagulation markers, 2 of blood lipid parameters and 0 of inflammatory markers (see Tables 1–5). With respect to the secondary literature search, we identified 6 potentially relevant publications after removal of duplicates and checking of formal inclusion criteria (see Figure 1). Only 1 of these 6 publications met content-related inclusion criteria. We additionally identified 1 eligible publication from reference lists, rendering a total of 2 articles included in the qualitative synthesis of literature on reactivity to AMS in hypertension-prone individuals with respect to PNS, HPA axis, RAAS, and intermediate biological risk factors.

**Table 1:** Summary of studies on stress reactivity to AMS in EHT with respect to SNS parameters.

Authors	SNS parameter	Stressor (duration in min)	Participant characteristics				Hypertension cut-offs	Results
			*n*	Age	Sex			
Increased reactivity to AMS of at least one SNS parameter in EHT								
Flaa et al. (98)	NEP EP MBP HR	MA (5)	13 high BP (166/97 ± 3/1 mmHg) 15 intermediate BP (129/79 ± 2/1 mmHg) 15 low BP (106/52 ± 2/2 mmHg)	19.0 ± 0	♂		.	NEP/EP/MBP/HR: + in high BP
Garafova et al. (99)	NEP EP SBP/DBP HR	SCWT (10)	8 EHT with BMI <25 kg/m^2^ (HT) 13 NT with BMI <25 kg/m^2^ (NT) 10 EHT with BMI >30 kg/m^2^ (HT-OB) 14 NT with BMI >30 kg/m^2^ (NT−OB)	23.0 ± 3.0 23.0 ± 5.0 28.0 ± 4.0 27.0 ± 5.0	♂		SBP 140–159 mmHg and/or DBP 90–99 mmHg	NEP: + EP/SBP/DBP/HR: = No reactivity differences between lean and obese HT
Kawabe et al. (100)	NEP EP SBP/DBP HR	MA (5)	11 EHT-I 26 EHT-II 12 NT	19.3 ± 0.2 19.3 ± 0.2 19.7 ± 0.3	♂		EHT-I: ≥ 140/90 mm Hg EHT-II: only SBP ≥ 140 mm Hg	NEP: + in EHT-I; = EHT-II vs. NT SBP/DBP/HR: + in EHT-I as compared to EHT-II and NT EP: = ; no significant stress-induced changes
Lenders et al. (101)	NEP EP SBP/DBP HR	MA (5)	70 EHT 41 NT	35.2 ± 9.9 34.8 ± 9.5 Age classes: 20–29, 30–39, 40–55 years	♂	♀	SBP 140–179 mmHg and/or DBP 90–109 mmHg	NEP: + in HT aged 20–29, not in other HT-groups SBP: + EP/DBP/HR: =
Lindvall et al. (102)	NEP EP SBP/DBP HR	MA (5)	14 EHT 14 NT	37.0	♂	♀	SBP 140–160 mmHg and DBP 95–105 mmHg	NEP: + EP: = SBP: = (relative and absolute changes) DBP: = (absolute changes)−(relative changes) HR: = (relative and absolute changes)
Matsukawa et al. (103)	NEP EP MBP HR	MA (2)	9 EHT 11 NT	22 ± 1 21 ± 1	♂		SBP >140 mmHg and/or DBP >90 mmHg	NEP/EP/MBP/HR: +
Perini et al. (104)	NEP EP	SCWT (8), rest (16), MA (4)	24 EHT 50 NT + PH 49 NT−PH	18–24	♂	♀	SBP 140–160 mmHg and/or DBP 90–100 mmHg	NEP: SCWT: = MA: + EP: SCWT: = MA: =
Eliasson et al. (105)	NEP EP SBP/DBP HR	SCWT (.)	33 EHT 16 BHT 17 NT	40.0 ± 2.0 36.0 ± 2.0 38.0 ± 2.0	♂	♀	EHT: >160/95 mmHg BHT: SBP 140–160 mmHg or DBP 90–95 mmHg	EP: EHT vs. NT: + BHT vs. NT: + EHT vs. BHT: = DBP: BHT vs. EHT: + in BHT BHT vs. NT: + in BHT EHT vs. NT: = NEP/SBP/HR: =
Palermo et al. (106)	EP	MA (5)	15 EHT 15 NT	39 ± 3	♂		.	EP: +
Tomoda et al. (107)	NEP EP SBP/DBP HR	MA (10)	11 EHT-I 13 EHT-II 14 NT	40 ± 4 49 ± 3 42 ± 4	♂	♀	EHT-I: SBP 130–139 mmHg and/or DBP 80–89 mmHg EHT-II: SBP >140 mmHg and/or DBP >90 mmHg	EP: EHT-I vs. NT: = EHT-II vs. NT: + SBP: EHT-I vs. NT: = EHT-II vs. NT: + HR: EHT-I vs. NT: = EHT-II vs. NT: + NEP: = DBP: =
Wirtz et al. (42)	NEP EP SBP/DBP HR	TSST (15)	22 EHT 26 NT	46.3 ± 3.0 42.0 ± 2.6	♂		SBP ≥ 140 mmHg and/or DBP ≥ 90 mmHg	EP: + NEP/SBP/DBP/HR: =
Bahlmann et al. (108)	MBP	MA (4)	12 EHT 6 NT	.	.	.	SBP 130–159 mmHg or DBP 80–94 mmHg	MBP: +
Baumann et al. (109)	NEP SBP/DBP HR	MA (14)	30 EHT 20 NT	15–20	♂		SBP 130–139 mmHg or DBP 80–89 mmHg	SBP/DBP/HR: + NEP: =
Brod et al. (110)	SBP/DBP HR	MA (5)	12 EHT 6 NT	35.6	♂	♀	140/90–179/109 mmHg	SBP/DBP: + HR: =
Drummond (111)	SBP/DBP HR	MA (6)	18 EHT 18 NT	25.0 25.2	♂	♀	SBP ≥ 140 mmHg and/or DBP ≥ 90 mmHg	SBP/DBP: + HR: =
Drummond (112)	SBP/DBP HR	MA (5)	16 EHT 10 NT	22.9 25.3	♂		SBP >140 mmHg	SBP: + DBP/HR: =
Fredrikson et al. (113)	SBP/DBP	MA (2)	14 EHT 14 NT	43.6 ± 2.5 44.2 ± 2.6	♂	♀	>160/95 mmHg	SBP: + DBP: =
Georgiades et al. (114)	SBP/DBP HR	MA (4)	75 EHT 74 NT	50.0 ± 5.7 49.9 ± 5.7	♂		DBP: 85–94 mmHg	SBP: + DBP/HR: =
Itoh et al. (115)	MBP HR	MA (10)	26 EHT 24 NT	30–59	♂		SBP ≥ 140 mmHg and DBP ≥ 90 mmHg	MBP: + in 30-year-olds; = in 40- and 50-year-olds HR: =
Köhler et al. (116)	SBP/DBP HR	MA (10)	Study-1: 19 HT 19 NT Study-2: 18 HT 18 NT	20.74 ± 1.19 20.89 ± 1.66 21.33 ± 2.15 21.28 ± 1.49	♂		SBP >140 mmHg and/or DBP >90 mmHg	SBP: + DBP/HR: =
Langewitz et al. (117)	SBP/DBP	MA (5)	34 HT 41 BHT 54 NT	44 ± 7	♂		HT: SBP ≥ 160 mmHg and/or DBP ≥ 95 mmHg BHT: SBP 140–159 mmHg and/or DBP 90–94 mmHg	SBP: + in HT as compared to BHT and NT DBP: + in HT and BHT as compared to NT
Lindqvist et al. (118)	NEP EP SBP/DBP HR	SCWT (10)	11 EHT 10 NT	40.5 38.8	♂		DBP 95–115 mmHg	SBP: + NEP/EP/DBP/HR: =
Reims et al. (119)	NEP EP SBP/DBP HR	MA (5)	20 EHT 20 NT	22.2 ± 0.6 22.7 ± 0.6	♂		SBP >140 mmHg and DBP >90 mmHg	DBP: + NEP/EP/SBP/HR: =
Schmieder et al. (120)	SBP/DBP HR	MA (5)	12 EHT 14 NT+PH 12 NT−PH	24 ± 3 24 ± 2 24 ± 2	♂		SBP 140–160 mmHg and DBP 90–95 mmHg	SBP: + in HT as compared to NT+PH and NT−PH; NT+PH vs. NT−PH: = DBP/HR: =
Schulte & Neus (121)	SBP/DBP	MA (5)	10 EHT 10 BHT 10 NT	32.5 ± 6.8 31.6 ± 7.7 31.9 ± 7.7	♂		.	SBP: + in EHT and BHT as compared to NT DBP: + of EHT as compared to BHT and NT
Schulte et al. (122)	SBP/DBP	MA (5)	20 EHT 13 NT	.	♂	♀	SBP 130–159 mmHg or DBP 80–94 mmHg	SBP: + DBP: =
Shapiro et al. (123)	MBP	SCWT (.)	35 EHT 33 NT	47.1 36.6	♂	♀	DBP ≥ 90 mmHg	MBP: +
Steptoe et al. (124)	SBP/DBP HR	SCWT (4)	12 EHT 12 NT	42.3 ± 2.7 42.8 ± 2.8	♂		145/90–175/105 mmHg	SBP/DBP: + HR: =
Tuomisto (125)	SBP/DBP HR	MA (4)	32 EHT 30 BHT 33 NT	40.4 ± 4.2	♂		HT: SBP ≥ 160 mmHg or DBP ≥ 95 mmHg BHT: 140–159 mmHg or DBP 90–94 mmHg	SBP/DBP: EHT vs. NT: + BHT vs. NT: + EHT vs. BHT: = HR: =
Markovic et al. (126)	SBP/DBP HR	Mirror tracing task (3), rest (6), PST (5)	132 EHT 627 NT	.	♂	♀	SBP ≥ 140 mmHg and/or DBP ≥ 90 mmHg	SBP/DBP: + (both tasks) HR: = (both tasks)
Murakami et al. (127)	NEP EP MBP HR	MA (3)	23 EHT-I 11 EHT-II 10 NT	48.0 ± 3.0 48.0 ± 4.0 47.0 ± 3.0	♂	♀	EHT-I: SBP 130–139 mmHg and/or DBP 80–89 mmHg EHT-II: SBP >140 mmHg and/or DBP >90 mmHg	MBP: + in HT groups as compared to NT NEP/EP/HR: =
Walther et al. (128)	sAA	TSST (15)	19 EHT 23 NT	46.8 ± 3.4 42.1 ± 2.9	♂		SBP ≥ 140 mmHg and/or DBP ≥ 90 mmHg	sAA: +
Comparable SNS reactivity to AMS between EHT and NT								
Fossum et al. (129)	NEP EP MBP HR	MA (5)	96 high BP 22 normal BP	20–21	♂		High BP: ≥140/90 mmHg	NEP/EP/MBP/HR: =
Graafsma et al. (130)	NEP EP SBP/DBP HR	MA (5)	20 EHT 20 NT	42.6 ± 7.5 46.0 ± 6.7	♂	♀	SBP >140 mmHg and/or DBP >90 mmHg	NEP/EP/SBP/DBP/HR: =
Hjemdahl & Eliasson (131)	NEP EP SBP/DBP HR	SCWT (20)	7 EHT 7 NT	34.6	.	.	.	NEP/EP: = ; no significant stress-induced changes SBP/DBP/HR: =
Januszewicz et al. (132)	NEP EP	MA (30)	10 EHT 10 NT	26.9 32.9	♂		.	NEP/EP: =
Köhler et al. (133)	NEP EP SBP/DBP HR	MA (10)	12 EHT 12 NT	21.00 ± 1.54 21.50 ± 1.51	♂		SBP >140 mmHg and/or DBP >90 mmHg	NEP/EP/SBP/DBP/HR: =
Carroll et al. (134)	SBP/DBP	MA (4)	12 HT 11 NT	22 ± 12.9 21 ± 25.2	♂		SBP ≥ 140 mmHg	SBP/DBP/HR: =
Esler & Nestel (135)	SBP/DBP	Visual puzzles (45)	10 EHT 6 NT	.	.	.	>145/90 mmHg	SBP/DBP: =
Fredrikson et al. (136)	SBP/DBP HR	MA (2)	14 EHT 14 NT	41.6 ± 8.7 44.3 ± 8.6	♂	♀	SBP ≥ 160 mmHg and/or DBP ≥ 95 mmHg	SBP/DBP/HR: =
Hollenberg et al. (137)	MBP HR	IQ test (20)	15 EHT 24 NT	40.1 ± 4.0 38.9 ± 3.9	.	.	.	MBP/HR: = ; no significant stress-induced changes
Naqvi & Hyuhn (138)	SBP/DBP HR	SCWT (3), MA (3), randomized order with 15 min rest	28 EHT 20 NT	51 ± 13 49 ± 11	♂	♀	SBP 136–180 mmHg or DBP 86–100 mmHg	SBP/DBP/HR: =
Nyklicek et al. (20)	SBP/DBP HR	MA (7), rest (5), PST (5)	37 EHT 20 NT	43.8 ± 6.0 44.8 ± 5.6	♂	♀	SBP ≥ 140 mmHg and/or DBP ≥ 90 mmHg	SBP/DBP/HR: =
Scheuch et al. (139)	SBP/DBP HR	MA (6)	17 EHT 17 NT	39.9 ± 14.2 37.1 ± 11.4	.	.	SBP > 130 mmHg and DBP > 80 mmHg	SBP/DBP/HR: =
Seibt et al. (140)	SBP/DBP HR	SCWT (6), rest (3), MA (6)	20 EHT 20 BHT 20 NT	34.4 ± 7.6 27.9 ± 5.7 28.0 ± 6.8	♂	♀	EHT: >18 of 30 recordings >140/90 mmHg or >6 of 30 recordings >160/95 mmHg BHT: all between HT and NT NT: <8 of 30 BP recordings >140/90 mmHg & 0 recordings >160/95 mmHg	SBP/DBP/HR: =
Decreased reactivity to AMS of at least one SNS parameter in EHT (and no increases in any SNS parameter)								
Ducher et al. (141)	SBP/DBP	SCWT (20)	10 EHT 10 NT+PH 10 NT−PH	39 ± 1 30 ± 3 42 ± 2	♂	♀	DBP >95 mmHg	SBP/DBP: HT vs. NT-PH: = HT vs. NT+PH: −
Horikoshi et al. (142)	NEP EP SBP/DBP HR	MA (3)	8 EHT 14 NT+PH 14 NT−PH	24.6 ± 4.3 21.9 ± 2.4 21.9 ± 2.6	♂	♀	SBP 140–160 mmHg and/or DBP 90–95 mmHg	DBP:−in EHT as compared to NT−PH and NT+PH NEP/EP/SBP/HR: =
Sullivan et al. (143)	NEP EP SBP/DBP HR	MA (.)	15 EHT 13 NT	37.0 ± 11.0 36.0 ± 8.0	♂	♀	SBP >140 mmHg and/or DBP >90 mmHg	SBP:−NEP/EP: = ; no significant stress-induced changes DBP/HR: =
Dimsdale et al. (144)	SBP/DBP HR	MA (3)	21 EHT 25 NT	39 ± 5 37 ± 6	.	.	SBP ≥ 140 mmHg and DBP ≥ 90 mmHg	HR:− SBP/DBP: =
Fredrikson et al. (145)	SBP/DBP HR	MA (2)	14 EHT 14 NT	46.5 ± 8.3 40.3 ± 9.9	♂	♀	SBP ≥ 160 mmHg and/or DBP ≥ 95 mmHg	HR:− SBP/DBP: =

BHT, borderline hypertensive individuals; BP, blood pressure; DBP, diastolic blood pressure; EHT, essential hypertensive individuals; EP, epinephrine; HR, heart rate; HT, hypertensive individuals; MA, mental arithmetic task; MBP, mean arterial blood pressure; NEP, norepinephrine; NT, normotensive individuals; NT + PH, NT with parental hypertension; NT−PH, NT without parental hypertension; PST, public speaking task; sAA, salivary alpha-amylase; SBP, systolic blood pressure; SCWT, stroop-color-word-conflict test; SNS, sympathetic nervous system; TSST, trier social stress test;., not reported;  + , hyperreactivity in HT;  = , similar reactivity; −, diminished reactivity in HT.

**Table 2:** Summary of studies on stress reactivity to AMS in EHT with respect to PNS parameters.

Authors	PNS parameter	Stressor (duration in min)	Participant characteristics				Hypertension cut-offs	Results
			*n*	Age	Sex			
Increased reactivity to AMS of at least one PNS parameter in EHT								
Langewitz et al. (117)	HF	MA (5)	34 HT 41 BHT 54 NT	44 ± 7	♂		HT: SBP ≥ 160 mmHg and/or DBP ≥ 95 mmHg BHT: SBP 140–159 mmHg and/or DBP 90–94 mmHg	HF: BHT vs. NT:+ HT vs. NT: +
Ruediger et al. (146)	HF	MA (6)	20 EHT 20 NT	30.5 ± 6.0 25.3 ± 6.0	♂		>135/85 mmHg	HF: +
Comparable PNS reactivity to AMS between EHT and NT								
Garafova et al. (99)	HF HFnu	SCWT (10)	8 EHT with BMI <25 kg/m^2^ (HT) 10 EHT with BMI >30 kg/m^2^ (HT-OB) 13 NT with BMI <25 kg/m^2^ (NT) 14 NT with BMI >30 kg/m^2^ (NT−OB)	23.0 ± 3.0 28.0 ± 4.0 23.0 ± 5.0 27.0 ± 5.0	♂		SBP 140–159 mmHg and/or DBP 90–99 mmHg	HF/HFnu: =; no significant stress-induced changes
Itoh et al. (115)	HF	MA (10)	26 EHT 24 NT	30–59	♂		SBP ≥ 140 mmHg and DBP ≥ 90 mmHg	HF: =; no significant stress-induced changes
Seipäjärvi et al. (147)	RMSSD	TSST–group version (26)	73 patients with cardiometabolic risk factors (EHT and/or prediabetes or type 2 diabetes) (PT) 63 young NT (HY) 61 middle-aged NT (HM)	53 ± 8 26 ± 3 52 ± 5	♂	♀	.	RMSSD: =

DBP, diastolic blood pressure; EHT, essential hypertensive individuals; HF, high frequency powers of heart rate variability; HFnu, high frequency powers of heart rate variability expressed in normal units; HT, hypertensive individuals; MA, mental arithmetic task; NT, normotensive individuals; PNS, parasympathetic nervous system; RMSSD, root mean square of successive differences; SBP, systolic blood pressure; SCWT, stroop-color-word-conflict test; TSST, trier social stress test; not reported.

**Table 3:** Summary of studies on stress reactivity to AMS in EHT with respect to HPA axis parameters.

Authors	HPA parameter	Stressor (duration in min)	Participant characteristics				Hypertension cut-offs	Results
			*n*	Age	Sex			
Increased cortisol reactivity to AMS in EHT or HT-prone individuals								
Baumann et al. (109)	CORT (plasma)	MA (14)	30 EHT 20 NT	15–20	♂		.	CORT: +
Nyklíček et al. (20)	CORT (salivary)	MA (7), rest (5), PST (5)	37 EHT 20 NT	43.8 ± 6.0 44.8 ± 5.6	♂	♀	SBP ≥ 140 mmHg and/or DBP ≥ 90 mmHg	CORT: +
Wirtz et al. (42)	CORT (salivary)	TSST (15)	22 EHT 26 NT	46.3 ± 3.0 42.0 ± 2.6	♂		SBP ≥ 140 mmHg and/or DBP ≥ 90 mmHg	CORT: +
Al’Absi & Wittmers (148)	CORT (salivary)	PST (24)	21 High Risk NT 26 Low Risk NT	18–59	♂	♀	High risk: above median resting SBP	CORT: + in high-risk individuals
Comparable cortisol reactivity to AMS between EHT and NT								
Hollenberg et al. (137)	CORT (plasma)	IQ Test (20)	15 EHT 24 NT	40.1 ± 4.0 38.9 ± 3.9	.	.	.	CORT: =; no significant stress-induced changes

ACTH, adrenocorticotropic hormone; BP, blood pressure; CORT, cortisol; DBP, diastolic blood pressure; EHT, essential hypertensive individuals; HPA axis, hypothalamus-pituitary-adrenal axis; MA, mental arithmetic task; NT, normotensive individuals; PST, public speaking task; SBP, systolic blood pressure; SCWT, stroop-color-word-conflict test; TSST, trier social stress test;., not reported; +, hyperreactivity in HT; =, similar reactivity; −, diminished reactivity in HT.

**Table 4:** Summary of studies on stress reactivity to AMS in EHT with respect to RAAS parameters.

Authors	RAAS parameter	Stressor (duration in min)	Participant characteristics				Hypertension cut-offs	Results
			*n*	Age	Sex			
Increased reactivity to AMS of at least one RAAS parameter in EHT								
Hollenberg et al. (137)	PRA ANG-II ALD	IQ Test (20)	15 EHT 24 NT	40.1 ± 4.0 38.9 ± 3.9	.	.	.	REN: + ANG-II: + ALD: +
Gideon et al. (149)	ALD	TSST (15)	21 EHT 25 NT	46.43 ± 3.15 41.76 ± 2.64	♂		SBP ≥ 140 mmHg and/or DBP ≥ 90 mmHg	ALD: +
Comparable RAAS reactivity to AMS between EHT and NT								
Baumann et al. (109)	PRA	MA (14)	30 EHT 20 NT	15–20	♂		.	REN: =
Dimsdale et al. (144)	PRA	MA (3)	21 EHT 25 NT	39 ± 5 37 ± 6	.	.	SBP ≥ 140 mmHg and DBP ≥ 90 mmHg	REN: =
Ducher et al. (141)	PRA ALD	SCWT (20)	10 EHT 10 NT+PH 10 NT−PH	39 ± 1 30 ± 3 42 ± 2	♂	♀	DBP >95 mmHg	REN:= ALD: =; no significant stress-induced changes
Esler & Nestel (135)	PRA	Visual puzzles (45)	10 EHT 6 NT	.	.	.	BP >145/90 mmHg	REN: =; no significant stress-induced changes
Hjemdahl & Eliasson (131)	PRA	SCWT (20)	7 EHT 7 NT	34.6	.	.	.	REN: =; no significant stress-induced changes
Tomoda et al. (107)	PRA ALD	MA (10 min)	11 EHT-I 13 EHT-II 14 NT	40 ± 4 49 ± 3 42 ± 4	♂	♀	EHT-I: SBP 130–139 mmHg and/or DBP 80–89 mmHg EHT-II: SBP >140 mmHg and/or DBP >90 mmHg	REN:= ALD: =

ALD, aldosterone; BP, blood pressure; DBP, diastolic blood pressure; EHT, essential hypertensive individuals; MA, mental arithmetic task; NT, normotensive individuals; NT+PH, NT with parental hypertension; NT−PH, NT without parental hypertension; PRA, plasma renin activity; RAAS, renin-angiotensin-aldosterone system; SBP, systolic blood pressure; SCWT, stroop-color-word-conflict test; TSST, trier social stress test; not reported; +, hyperreactivity in HT; =, similar reactivity; −, diminished reactivity in HT.

**Table 5:** Summary of studies on stress reactivity to AMS in EHT with respect to intermediate biological risk factors for cardiovascular disease.

Authors	Risk parameter	Stressor (duration in min)	Participant characteristics				Hypertension cut-offs	Results
			*n*	Age	Sex			
Increased reactivity to AMS of at least one risk parameter in EHT								
Palermo et al. (106)	Tissue plasminogen activator Tissue plasminogen antigen	MA (5)	15 EHT 15 NT	39 ± 3	♂		.	Tissue plasminogen antigen:+ Tissue plasminogen activator: −
Tomoda et al. (107)	Primary aggregation to reagents ß-thromboglobulin level ADP threshold for biphasic aggregation (ADP)	MA (10)	11 EHT-I 13 EHT-II 14 NT	40 ± 4 49 ± 3 42 ± 4	♂	♀	EHT-I: SBP 130–139 mmHg and/or DBP 80–89 mmHg EHT-II: SBP >140 mmHg and/or DBP >90 mmHg	Primary aggregation to reagents: EHT-I vs. NT:+ EHT-II vs. NT: =
								ß-thromboglobulin level: EHT-I vs. NT:+ EHT-II vs. NT: +
								ADP threshold: EHT-I vs. NT:− EHT-II vs. NT: =
Wirtz et al. (75)	FVII:C FVIII:C Fibrinogen D-Dimer	TSST (15)	21 systolic HT (SHT; with or without DHT) 22 diastolic HT (DHT; with or without SHT) 17 NT	44.2 ± 14.8 46.0 ± 12.4 41.0 ± 14.3	♂		SBP ≥ 135 mmHg and/or DBP ≥ 85 mmHg	FVII:C:+in DHT FVIII:C:+in DHT D-Dimer:+in DHT Fibrinogen: =
Wirtz et al. (13)	TC LDL-C HDL-C TG	TSST (15)	22 EHT 23 NT	46.3 ± 3.0 44.6 ± 2.4	♂		SBP ≥ 140 mmHg and/or DBP ≥ 90 mmHg	TC:+ LDL-C:+ HDL-C:= TG: =
Degroote et al. (81)	TC LDL-C HDL-C TG TC/HDL-ratio	MIST (30)	28 EHT 28 NT	49.82 ± 2.04 49.75 ± 2.22	♂		SBP ≥ 140 mmHg and/or DBP ≥ 90 mmHg	TC/HDL-ratio:+ TC:= LDL-C:= HDL-C:=TG:-
Comparable reactivity to AMS in risk parameters between EHT and NT								
Von Känel et al. (150)	Thrombin–antithrombin III complex (TAT) D-Dimer	PST (6), rest (15), Mirror Tracking Task (3)	6 HT 13 NT	39 ± 5	♂	♀	SBP >140 mmHg and/or DBP >90 mmHg	TAT:= D-Dimer: =

DBP, diastolic blood pressure; EHT, essential hypertensive individuals; FVII: C, blood clotting factor VII; FVIII:C, blood clotting factor VIII; HDL-C, high-density-lipoprotein cholesterol; HT, hypertensive individuals; LDL-C, low-density-lipoprotein cholesterol; MA, mental arithmetic task; MIST, Montreal Imaging Stress Test; NT, normotensive individuals; PST, public speaking task; SBP, systolic blood pressure; TC, total cholesterol; TG, triglycerides; TSST, trier social stress test; not reported; +, hyperreactivity in HT; =, similar reactivity; −, diminished reactivity in HT.

In the following, we report the results of our systematic review for the defined physiological parameters. For a condensed summary of our results, see Table 6.

**Table 6:** Summary of the findings of the systematic review.

		SAM axis					PNS		HPA axis		RAAS			Biological risk factors		
		NEP	EP	BP	HR	sAA	HF	RMSSD	ACTH	CORT	REN	ANG-II	ALD	INFL	COAGU	LIP
Stressor intensity	Mild	= (+ in young HT)	=	= (+ MA of 4–15 min length)	= (+ in young HT)	n.a	?	n.a.	n.a.	+	=	+	=	+	?	n.a.
	Moderate	n.a.	n.a.	?	=	n.a.	n.a.	n.a.	n.a.	+	n.a.	n.a.	n.a.	n.a.	=	n.a.
	Strong	=	+	=	=	+	n.a.	=	n.a.	+	n.a.	n.a.	+	n.a.	+	+

SAM axis, sympathetic adrenal medullary axis; EP, epinephrine; NEP, norepinephrine; BP, blood pressure; HR, heart rate; sAA, salivary alpha-amylase; PNS, parasympathetic nervous system, HF, high frequency powers of heart rate variability; RMSSD, root mean square of successive differences; HPA axis, hypothalamus-pituitary-adrenal axis; ACTH, adrenocorticotropic hormone; CORT, cortisol; RAAS, renin-angiotensin-aldosterone system; REN, renin; ANG-II, angiotensin-II; ALD, aldosterone; INFL, inflammatory parameters; COAGU, coagulation parameters; LIP, blood lipid parameters; +, existing literature pointing to a hyperreactivity in essential hypertension; =, existing literature pointing to similar reactivity in essential hypertensive and normotensive individuals;?, results of existing literature unclear; n.a., not applicable due to missing investigations.

#### 2.2.1. Stress reactivity of the autonomic nervous system

##### 2.2.1.1. Sympathetic adrenal medullary (SAM) axis

With respect to sympathetic parameters or SAM axis parameters, respectively, reactivity to AMS has been extensively studied in EHT. Given the multitude of studies, we restricted our review to studies that investigated sympathetic stress reactivity in manifest HT. We hereby focused our literature search on catecholamines, cardiovascular reactivity in terms of BP and HR as the major sympathetic measures, and sAA (see Table 1).

###### 2.2.1.1.1. Catecholamine reactivity

Norepinephrine: In reaction to AMS including either mild or strong mental stressors, the majority of studies failed to find reactivity differences in plasma NEP between HT and NT (42, 105, 107, 109, 118, 119, 127, 129–133, 142, 143) despite the overall higher plasma NEP levels with EHT (42, 142, 143). Notably, the participants of these studies were mostly aged between 35 and 50 years of age. However, few studies investigated reactivity to mild AMS by means of MA in younger subjects mostly aged between 19 and 29 (98–104). Results of these studies show enhanced NEP reactivity in the young HT as compared to NT. Interestingly, Lenders et al. (101) explicitly examined potential age-related differences in MA stress-induced plasma catecholamine reactivity in EHT. In line with the above summarized findings, they found increased NEP responses to MA in young HT aged 20–29 years, but not in older HT aged 30–39 or 40–55 years, all as compared to NT. These findings are in accordance with the hypothesis that in particular young HT and those in the early stages of hypertension are characterized by a hyperkinetic state with elevations in HR and cardiac output as well as increased sympathetic tone (151, 152). This hyperkinetic state is supposed to attenuate with increasing age and the progression of hypertension (151). In later stages of hypertension, BP elevations can be achieved with less sympathetic firing/tone because of alterations in the wall-lumen ratio and hyperresponsiveness of arterioles. This results in the combination of normal cardiac output and increased vascular resistance; characteristic for advanced hypertension (151, 153).

Epinephrine: Similar to NEP reactivity, the majority of studies did not observe differences in plasma EP reactivity to AMS between HT and NT (99–102, 104, 118, 119, 127, 129–133, 142, 143), apart from overall higher plasma EP levels with hypertension in some studies (99, 142). Notably, all these studies applied mild mental stressors. So far, only five studies report increased EP reactivity to AMS in HT with four studies employing mild mental stressors (98, 103, 105–107) and one study applying a strong stressor (42). To the best of our knowledge, the latter study is the hitherto only study investigating EP reactivity to a strong mental stressor. In that study, middle-aged EHT and NT men underwent the TSST, and EP reactivity was assessed repeatedly before and up to 60 min after stress cessation. Results showed higher EP reactivity in EHT as compared to NT with most pronounced reactivity differences up to 10 min after stress cessation pointing to EP hyperreactivity in reaction to strong AMS in middle-aged EHT. With respect to mild stressors, two studies investigated EP reactivity in younger subjects with an average age of 20 years to 2–5 min of MA and found higher EP reactivity in HT as compared to NT (98, 103) while other comparable studies did not (100, 104, 119, 129, 142). Notably, investigating EP reactivity in young men in the low, normal, and high (hypertension grade 2) BP ranges, Flaa et al. (98) found increasing EP reactivity with increasing BP. The last study investigated EP reactivity to the SCWT in two groups of EHT individuals, including grade 2 HT, aged from 18 to 59 years as compared to NT (105). In this study, the SCWT induced significant EP increases in the HT groups but not in NT. Other studies on EP reactivity to the SCWT, notably in HT grade 1, failed to find reactivity differences (99, 104, 118, 131). It remains to be elucidated whether an elevated EP reactivity to mild AMS may depend on HT severity.

###### 2.2.1.1.2. Cardiovascular reactivity

Blood pressure: In the majority of studies on BP reactivity to AMS, HT and NT exhibited similar SBP (20, 42, 99, 102, 105, 119, 130, 131, 133–136, 138–142, 144, 145), DBP (20, 42, 99, 101, 102, 107, 112–114, 116, 118, 120, 122, 130, 131, 133–136, 138–141, 143–145), and/or mean BP (MBP) (129, 137) stress reactivity with HT having comparatively higher overall BP levels. In contrast, comparably fewer studies report elevated SBP (100, 101, 107, 109–114, 116–118, 120–122, 124–126), DBP (100, 105, 109–111, 117, 119, 121, 124–126), and/or MBP (98, 103, 108, 115, 123, 127) reactivity to AMS in EHT. A more detailed look on methodological differences between the studies revealed that elevated SBP (100, 101, 107, 109–112, 114, 116, 117, 120–122, 125), DBP (100, 109–111, 117, 119, 121, 125), or MBP reactivity (98, 108, 115) was predominantly observed in studies using MA with a duration between 4 and 15 min. Neither studies using MA of shorter duration [increased BP reactivity in HT (103, 113, 127); similar BP reactivity (112, 113, 136, 138, 142, 144, 145); decreased BP reactivity in HT (142)], nor other similarly mild mental stressors [increased BP reactivity in HT (105, 118, 123, 124, 126); similar BP reactivity (99, 105, 118, 131, 135, 137, 140, 141)], nor stronger stress induction tasks [increased BP reactivity in HT: (126); similar BP reactivity (20, 42)]: were capable of reliably inducing higher BP reactivity in HT as compared to NT. Moreover, there was no clear evidence for effects of age or stressor severity (i.e., mild vs. moderate vs. strong) on BP stress reactivity in HT as have been associated with catecholamine reactivity (see above). Taken together, these findings suggest that elevated BP reactivity in HT seems to specifically relate to MA of a minimum length. Given that HT did not show a higher BP reactivity to other cognitive or stronger mental stressors, it might be speculated that the stressfulness of a task does not account for BP reactivity differences in HT. Whether the BP reactivity difference in HT relates to cognitive processes and related brain activity or psychophysical demands associated with MA or other mild mental stressors (e.g., SCWT) remains to be elucidated (154–156).

Heart rate: With respect to HR reactivity, the majority of studies could not reveal significant reactivity differences either to mild (20, 42, 99, 101, 102, 105, 110–112, 114–116, 118–120, 124–127, 129–131, 133, 134, 136–140, 142, 143, 145), moderate (20, 126), or strong (42) AMS between HT and NT. Increased HR reactivity to mild AMS in terms of MA in HT was observed in five studies that were all performed in younger HT (98, 100, 103, 107, 109). This further supports the above outlined hyperkinetic state in young HT and those in the early stages of hypertension (151, 152) (see Catecholamine reactivity). Moreover, one study reports increased HR reactivity to MA in individuals with hypertension grade 2 but not with hypertension grade 1 as compared to NT (107). Of all studies only two studies found diminished HR reactivity in reaction to MA in middle-aged EHT (144, 145). Taken together, there is weak evidence for a HR hyperreactivity to AMS in EHT, except for a subgroup of hyperkinetic HT.

###### 2.2.1.1.3. Salivary alpha amylase

In the hitherto only study investigating sAA reactivity to AMS in EHT, we recently found that HT exhibit greater sAA reactivity to the TSST as a strong mental stressor as compared to NT with higher sAA increases +1 min and +10 min after TSST cessation (128). This suggest that the stress-induced physiological hyperreactivity in EHT also extends to the SNS parameter sAA.

##### 2.2.1.2. Parasympathetic nervous system (PNS)

So far, PNS reactivity to AMS in manifest EHT without further comorbidity has been investigated in four studies that applied mild mental stressors and assessed the PNS parameter HF (see Table 2). Two of the studies could not find significant stress-induced HF changes, neither in NT nor in HT to MA (115) or SCWT (99). However, the other two studies observed greater HF decreases in HT as compared to NT in reaction to MA pointing to a reduced parasympathetic cardiac control during stress in HT (117, 146). In line with the latter, a recent study investigated the RMSSD response to the TSST as strong mental stressor in patients with cardiometabolic risk factors, i.e., with EHT and/or either prior evidence of prediabetes or type-2 diabetes as compared to healthy NT (147). Patients with cardiometabolic risk factors had overall lower RMSSD values during rest as well as during stress. PNS reactivity to AMS in hypertension-prone individuals has not yet been investigated. Taken together, these studies may suggest that the reduced parasympathetic cardiac control under resting conditions (157, 158) seem to extend to AMS, in terms of a greater reduction of PNS activity under stress, most likely dependent on the intensity of the stressor.

Taken together, the above summarized findings regarding stress reactivity of the autonomic nervous system suggests that SAM axis hyperreactivity cannot generally be observed in HT but occurs only in response to specific mental stressors or in an early hyperkinetic stage of HT (see Table 6).

#### 2.2.2. Hypothalamus pituitary adrenal (HPA) axis

Regarding HPA axis reactivity to AMS in human EHT (see Tables 3, 6), previous studies focused on the HPA axis stress hormone cortisol whereas ACTH has hitherto not been investigated in EHT. Compared to NT, HT exhibited larger elevations in plasma (109, 148) as well as salivary cortisol (20, 42) during and after mild, moderate, and strong mental stress, however not unequivocally (137). Also, hypertension-prone individuals have been reported to show higher salivary cortisol responses to moderate mental stress (148). There are hitherto no studies investigating ACTH reactivity to AMS in hypertension-prone individuals. Notably, animal studies point to an HPA axis hormone-spanning stress hyperreactivity with hypertension (159, 160). Whether the heightened HPA axis stress reactivity in human EHT already occurs at the level of the pituitary gland where ACTH is secreted from the portal system into the circulation, remains to be elucidated. Moreover, given the observed prospective association between higher cortisol reactivity to AMS and incident hypertension (6), it can be assumed that increased HPA axis reactivity is present before the onset of hypertension. Thus, the magnitude of HPA axis stress reactivity may constitute one possible mechanism through which acute stress may influence the risk of hypertension and CVD (4, 6).

#### 2.2.3. Renin-angiotensin-aldosterone system

In the context of human hypertension, reactivity of RAAS parameters to AMS has so far hardly been investigated (see Table 4). With respect to mental stress-induced *plasma renin* reactivity, studies applying mild AMS could not find significant differences between HT and NT (107, 109, 131, 135, 141, 144). Notably three of them found negligible PRA changes in both, HT and NT (131, 135, 144) and three observed slightly but not significantly higher PRA increases in HT as compared to NT (107, 109, 141). In response to a mild stress-inducing IQ-Test however, HT exhibited increases in PRA as compared to the decreases observed in the NT (137). This study is also the only study investigating RAAS reactivity to AMS in hypertension-prone individuals. Hypertension-prone individuals showed PRA increases in-between NT and HT (137). Moreover, this study is also the hitherto only one considering *ANG-II* reactivity to AMS so far, revealing a similar reactivity pattern for ANG-II as observed for PRA, with increases in the hypertension-group, decreases in the normotensive-group, and hypertension-prone individuals to be in-between (137). So far, renin or ANG-II reactivity to stronger AMS induction procedures have not yet been investigated in or hypertension-prone individuals as compared to NT. With respect to *plasma aldosterone* reactivity, two of the three studies applying mild mental stressors, did not reveal reactivity differences between HT and NT in reaction to the SCWT (141) or in reaction to MA (107). In the second study however, HT showed stress-induced aldosterone increases after an IQ-test significantly differing from the decreases observed in NT (137). We recently investigated aldosterone reactivity in EHT, applying the TSST as a strong psychosocial stressor and overserved significantly greater aldosterone increases in EHT as compared to NT (149). There are hitherto no studies investigating aldosterone reactivity to AMS in hypertension-prone individuals.

Taken together, in reaction to mild AMS induction, results are mixed but point to a (slightly) higher reactivity of all RAAS parameters in hypertension, whereas strong AMS induction induced significantly higher reactivity of the RAAS end-product aldosterone in EHT (see Table 6). Overall, these results suggest that the stress-induced physiological hyperreactivity in EHT also extends to the RAAS depending on the intensity of the stressor.

#### 2.2.4. Intermediate biological risk factors for cardiovascular disease

##### 2.2.4.1. Coagulation activity

So far, four studies investigated coagulation activity in response to AMS in HT as compared to NT (see Table 5). In terms of mild mental stress, one study observed attenuated fibrinolysis activation in terms of t-PA reactivity (106) and another study found greater platelet activity (107) in HT as compared to NT in response to MA. A third study applied a speech task and a mirror tracking task in randomized order as mild and moderate mental stressors, respectively, and could not find group differences in stress-induced increases in TAT and D-Dimer, notably in a rather small sample of 6 HT and 13 NT (150). There is one study examining coagulation reactivity in EHT in reaction to strong mental stress (75). In this study, HT exhibited exaggerated acute procoagulant responses as compared to NT. These responses became most apparent during recovery of hypercoagulability from stress depending on hypertension subtype with diastolic HT showing higher FVII:C levels immediately post-stress and at 20-min recovery, higher FVIII:C levels at 20- and 60-min recovery as well as higher D-dimer recovery (75). Coagulation activity in response to AMS in hypertension-prone individuals as compared to NT has hitherto not been investigated. Taken together, there is compiling evidence for an exaggerated coagulation reactivity to AMS in EHT, especially in diastolic HT, that seems to be most apparent during recovery from prothrombotic changes from stress (see Table 6).

##### 2.2.4.2. Blood lipids and lipoproteins

Blood lipid reactivity to AMS has hardly been investigated in EHT. There are hitherto no studies investigating blood lipid reactivity in reaction to mild or moderate AMS in EHT, however, two studies investigated blood lipid reactivity in response to strong mental stress (13, 81) (see Table 5). In reaction to the TSST, hypertensive men exhibited greater TC and LDL-C responses as compared to normotensive controls, with group differences sustaining up to 60 min after TSST cessation and most pronounced differences immediately after TSST cessation (13). There were no reactivity differences in HDL-C and TG. In reaction to the MIST, notably performed within an fMRI environment in lying position wearing goggles, HT showed a more pronounced rise in TC/HDL-C ratio scores and a slower decrease in TG levels as compared to NT but no reactivity differences in TC, HDL-D, and LDL-C (81). Notably, complementary analyses using MAP as continuous measure of hypertension assessment confirmed the linear nature of the observed effects in both studies. Whether the observed reactivity differences in some of the lipid markers are generalizable and relate to differences between the TSST and the MIST, e.g., such as perceived stressor intensity, remains to be elucidated. There are no studies investigating blood lipid reactivity in response to AMS in hypertension-prone individuals.

Taken together, the above outlined studies indicate an exaggerated blood lipid stress reactivity with hypertension, at least to strong mental stress (see Table 6). Whether this extends to mild mental stressors remains to be elucidated.

##### 2.2.4.3. Inflammatory markers

So far, there are no studies investigating reactivity of inflammatory markers to AMS in human EHT or in hypertension-prone individuals (see Table 5). Notwithstanding, there is few evidence pointing to a potentially exaggerated inflammatory responses to AMS in EHT. Two studies in manifest HT investigated stress reactivity of immune parameters related to secretion of inflammatory markers (20, 161). In reaction to mild mental stress, HT exhibited a circulatory environment conducive to increased leukocyte adhesion including higher increases in T-lymphocytes (161), both central to atherogenesis, as well as higher levels of the antibody type immunoglobulin A measured from saliva (20) as compared to NT controls. Moreover, studies investigating associations between inflammatory markers and BP instead of EHT, similarly point to a potentially exaggerated inflammatory stress response with increasing BP and thus EHT. Two cross-sectional studies, notably in NT, report associations between cardiovascular and inflammatory reactivity to mild mental stress (162, 163). Increasing BP stress reactivity was found to be associated with higher levels of IL-6 and IL-1 receptor antagonist (162) as well as higher stress-induced IL-1β gene expression (163).

To sum it up, differences in inflammatory reactivity to AMS between HT and NT have not been investigated so far (see Table 6). However, results from studies investigating stress reactivity of immune parameters in HT or associations between inflammatory stress reactivity and BP stress reactivity suggest that elevated stress reactivity of inflammatory markers might relate to EHT, in particular prospectively.

## 3. Potential mechanisms underlying physiological hyperreactivity to AMS in essential hypertension

Potential mechanisms underlying the above summarized physiological hyperreactivity to AMS in EHT have conceptually been divided into three different systemic levels of influence (164): the cognitive-emotional level, the hypothalamic and brainstem level, and the peripheral level. Alterations in physiological stress reactivity with EHT may arise from systematic differences at the *cognitive-emotional level* encompassing perception and evaluation of and affective responses to (mental) stressors as well as the concomitant cortical processing. Notably, these processes depend on the activity of the corticolimbic system and are important determinants of the physiological reactivity. Differences in physiological stress reactivity may (additionally or alternatively) emerge at the *hypothalamic and brainstem level*, where inputs from the corticolimbic systems are converted into autonomic or neuroendocrine (re)activity (39). Last, stress reactivity differences may result from tissue alterations at the *peripheral level*, such as alterations in vascular or receptor functioning.

### 3.1. Cognitive-emotional level

Various *psychological factors* have been proposed to affect the perception and evaluation of a stressor as well as the affective response that may contribute to the tonic elevation of sympathetic drive and to the frequent and intense bouts of stressor-evoked reactivity observed in HT (164, 165). These psychological factors include social support, affect management or emotional regulation respectively, chronic stress, in addition to cognitive appraisal of the stress situation (165). Interestingly, differences between HT and NT have repeatedly been observed with respect to some of these variables with HT reporting lower levels of social support and higher levels of perceived chronic stress (42, 166–168). They were found to differ in emotional regulation strategies, in particular with deficits in positive strategies, but interestingly not in the cognitive appraisal of a standardized stressor (42). Despite these observed differences, the role of psychological factors in physiological stress reactivity in HT has hardly been investigated so far. The hitherto only study investigating associations of psychological factors with physiological AMS reactivity in EHT examined associations of social support, hedonistic emotional regulation (HER), and cognitive appraisal with endocrine stress reactivity to strong mental stress (42). With respect to *social support*, HT with lower perceived social support showed higher EP reactivity to the TSST as compared to NT and HT with higher perceived social support. With respect to *HER*, HT with lower HER exhibited higher NEP reactivity and higher cortisol reactivity to the TSST as compared to NT and HT with high HER. In sum, findings of that study suggest a possible role for social support and emotional regulation in modulating endocrine stress reactivity in EHT. Whether such modulated endocrine stress reactivity in turn affects intermediate biological CVD risk factors and whether such a mechanism may link HT with poor cardiovascular outcomes remains to be elucidated. With respect to *chronic stress*, some studies in NT found increased cardiovascular or neuroendocrine reactivity with higher chronic stress (169, 170), but the role of chronic stress in physiological reactivity to AMS in EHT has not yet been investigated.

In addition to psychological factors, the cognitive emotional level is affected by the *neural structure, function, and connectivity* of the corticolimbic system (171). In particular, alterations of the corticolimbic system may relate to the stress reactivity differences with EHT (172). In reaction to mild acute stress, HT exhibited altered (re)activity in corticolimbic brain areas proposed to be involved in stress regulation (39, 173), including prefrontal and cingulate cortex, insula, hippocampus, as well as the amygdala (174–176). While imaging studies that additionally assess physiological stress reactivity are lacking in HT, evidence from studies in NT points to a neural basis of elevated cardiovascular and cortisol stress reactivity (173, 177, 178). Future studies are needed to test for a potential neural basis of the observed elevated cardiovascular and cortisol stress reactivity in EHT.

### 3.2. Hypothalamic and brainstem level

Multiple functional alterations of subcortical structures including hypothalamus, medulla, and pons, have been observed in HT and may relate to the stress reactivity differences with EHT (179, 180). For instance, increased NEP release from subcortical brain regions has been observed in HT as compared to NT (181). This finding is in line with animal studies investigating brain catecholamines and points to a disturbance of catecholaminergic metabolism in the inferior part of the brain in EHT (179, 182, 183). Further, functioning of the hypothalamic paraventricular nucleus involved in sympathetic and parasympathetic control of cardiovascular reactivity, have been proposed to be altered with HT (184, 185). Moreover, investigating CRH-producing neurons of the paraventricular nucleus using post-mortem quantitative immunohistochemical and *in situ* hybridization techniques, higher numbers of CRH-producing neurons and higher amount of CRH mRNA have been found in EHT as compared to healthy controls (186). Given the central role of CRH in the activation the HPA axis but also in the activation of the SNS (187), the suggested elevated CRH synthesis and release may contribute to the hyperreactivity of the SNS and the HPA axis. However, investigation of associations between stress reactivity on the cerebral level with endocrine and/or cardiovascular stress reactivity in EHT so lacking so far.

### 3.3. Peripheral level

At the peripheral level, we investigated whether stress hormone reactivity in terms of NEP, EP, and cortisol, contributes to blood lipid and lipoprotein or procoagulant stress reactivity with elevated BP (12, 13). We assessed plasma NEP, EP, cortisol as well as FVII:C, FVIII:C, fibrinogen, D-dimer, and plasma lipid profiles before and +1, +20, and +60 min after cessation of the TSST in hypertensive men as compared to NT (12, 13). We additionally considered the influence of resting mean arterial BP (MAP) as a continuous indicator of hypertension status.

With respect to *blood lipid parameter reactivity*, we observed that stress-induced NEP increases independently predicted higher immediate stress increases in TC, TG, and LDL-C (13). We hypothesized this association to result from NEP induced lipolysis and release of free fatty acids which in turn may serve as a substrate for the resynthesis of TG and hepatic production of very-low-density lipoprotein cholesterol (13, 86). Moreover, MAP interacted with NEP increases in predicting immediate changes in HDL-C and LDL-C (13). Given this, NEP stress reactivity seems to elicit proatherogenic changes of blood lipids and lipoproteins in response to AMS, either alone or in interaction with MAP, pointing to a further potential mechanism by which stress might increase cardiovascular risk in HT.

With respect to *coagulation activity*, we observed that NEP changes from rest to immediately after stress independently predicted immediate D-Dimer changes (12). Also, cortisol changes from rest to 10 min after stress independently predicted immediate FVIII:C changes. In terms of associations between integrated reactivity of stress hormone and coagulation factors between rest and 60 min after stress cessation (total stress reactivity), MAP interacted with the integrated release of EP in predicting total stress reactivity of FVII:C and of fibrinogen. MAP further interacted with integrated NEP release in predicting total FVIII:C stress reactivity while D-dimer total stress reactivity was predicted by NEP stress reactivity alone. These findings suggest a sustained hypercoagulability over about 1 h after stress in EHT which may represent a psychobiological mechanism through which stress and hypertension may interact in increasing the risk of atherothrombotic events (188).

Moreover, alterations in adrenergic receptor (AR) number and/or functioning have been observed with EHT (189–191). Notably, these AR alterations may add to the physiological reactivity differences in EHT. With respect to cardiovascular reactivity, β-AR mechanisms do not seem to mediate the higher reactivity in HT as compared to NT (192–194), but we recently found evidence pointing to a role for α-AR mechanisms (195). We investigated α-AR mechanisms in the cardiovascular reactivity to a standardized stress reactivity-mimicking NEP-infusion in EHT as compared to NT by using the non-selective α-AR blocker phentolamine. Non-selective α-AR blockade attenuated DBP hyperreactivity to NEP-infusion observed in HT but not SBP hyperreactivity in HT, suggesting a mediating effect of α-AR in DBP hyperreactivity to stress in EHT. Whether alterations in α- and/or β-AR number and/or functioning is involved in the observed hyperreactivity of other stress-reactive parameters in EHT remains to be elucidated.

**Figure 2:**
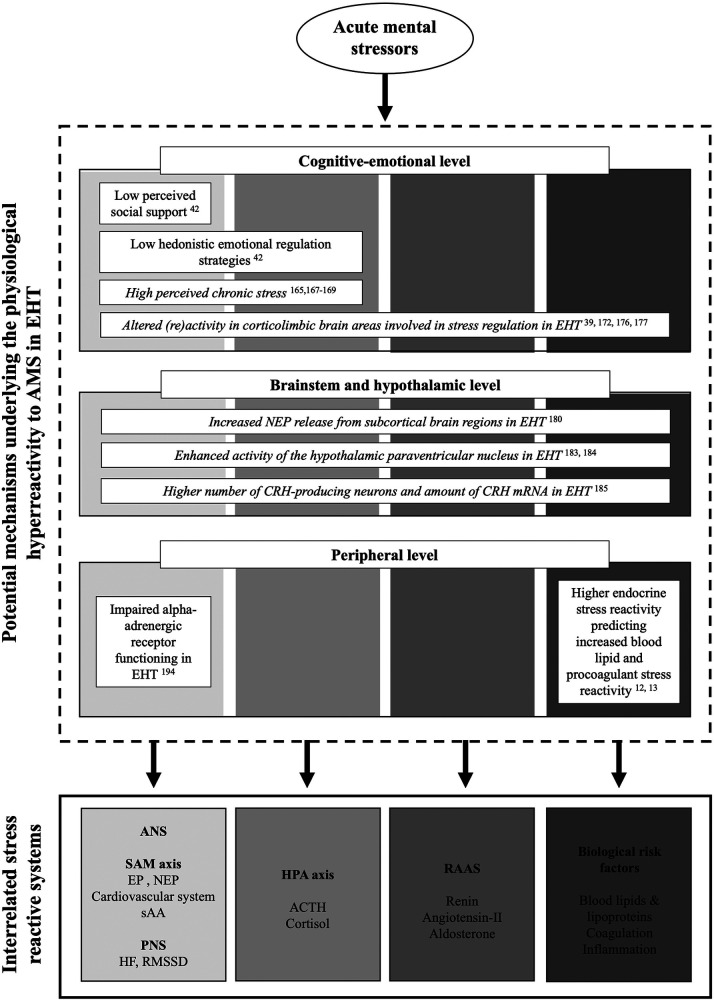
Overview over potential mechanisms underlying the physiological hyperreactivity to acute mental stress in essential hypertension with respect to the cognitive-emotional, brainstem and hypothalamic, as well as peripheral levels and the different stress reactive physiological systems. Potential mechanisms with respective evidence are presented in regular font. Potential suggested mechanisms but not yet investigated in the context of acute stress reactivity are presented in italics.

In sum, there are several potential mechanisms operating at different levels that may contribute to the altered physiological reactivity to AMS with EHT (for summary see Figure 2). However, the exact mechanisms and their interactions are not fully understood and need to be investigated in more detail.

## 4. Conclusion and future directions

In this comprehensive systematic review, we summarized the hitherto published evidence on increased physiological reactivity to AMS in EHT as compared to NT with respect to the SAM axis, the HPA axis, the RAAS, as well as stress-reactive intermediate biological risk factors for CVD. Taken together, our review indicates that the expected physiological hyperreactivity to AMS cannot generally (i.e., to mental stressors of all intensities, across all stress-reactive physiological systems, and in all HT) be observed in EHT. In fact, in reaction to *acute mental stressors of mild or moderate intensity*, as applied by most studies investigating physiological, in particular sympathetic, reactivity to AMS in EHT, results were inconclusive, presumably due to methodological issues. More precisely, our review indicates that exaggerated reactivity to mild AMS occurs only in response to mild mental stressors with specific characteristics, in an early hyperkinetic stage of HT, or with respect to certain stress systems. However, in reaction to *acute mental stressors of strong intensity*, such as the TSST or the MIST, evidence strongly suggests a physiological hyperreactivity across the investigated stress-reactive physiological systems. Our review revealed that there is considerable variation in the number of studies investigating reactivity to AMS in EHT with respect to the reviewed physiological systems. While reactivity of SAM axis parameters has been extensively studied, there are comparatively few studies examining reactivity to AMS with respect to PNS, HPA axis, or RAAS as well as with respect to reactivity of biological risk factors, pointing to the need for future research. Further, given that the first studies on physiological stress reactivity in EHT were published in the 1970s, the studies included in this review employed a strikingly broad methodology including statistical analysis methods. Future research replicating previous findings or further investigating differences in stress reactivity between HT and NT should apply state-of-the-art methods for hypertension classification, AMS induction, assessment of physiological parameters, as well as statistical analyses. As a limitation, it should be noted that the majority of studies included in our systematic review and the discussion of potential underlying mechanisms of stress hyperreactivity in EHT are cross-sectional studies and do not demonstrate a potential causal role with respect to hypertension severity or cardiovascular risk. Moreover, systematic reviews always entail the risk of publication bias, which means that only studies with significant results have been published.

The mechanisms underlying the observed differences in physiological reactivity to AMS between HT and NT are beginning to be understood and warrant further study. We discussed several potential mechanisms that may contribute to the observed reactivity differences at different levels, the cognitive-emotional level, the hypothalamic and brain stem level, and the peripheral level. Notably, the discussed potential mechanisms mainly come from study findings in samples of healthy NT participating in laboratory experiments and population-based studies, respectively. Further elucidation of the mechanisms in HT as compared to NT subjects is of vital importance, especially with regard to potential therapeutical targets for cardiovascular risk prevention and/or reduction. Concomitantly, potential modulating factors of the physiological hyperreactivity to AMS with EHT remain to be elucidated.
